# Highly Elevated Quantitative D-Dimer Assay Values Increase the Likelihood of Venous Thromboembolism

**DOI:** 10.1055/s-0038-1677029

**Published:** 2019-01-07

**Authors:** Samuel Francis, Alexander Limkakeng, Hui Zheng, Judd Hollander, Gregory Fermann, Blair Alden Parry, Frank Lovecchio, Nikos Werner, Sebastian Schellong, Christopher Kabrhel

**Affiliations:** 1 Division of Emergency Medicine, Duke University Hospital, Durham, North Carolina, United States; 2 Department of Biostatistics, Massachusetts General Hospital, Boston, Massachusetts, United States; 3 Department of Emergency Medicine, Jefferson University, Philadelphia, Pennsylvania, United States; 4 Department of Emergency Medicine, University of Cincinnati, Cincinnati, Ohio, United States; 5 Department of Emergency Medicine, Center for Vascular Emergencies, Massachusetts General Hospital, Boston, Massachusetts, United States; 6 Department of Emergency Medicine, Maricopa Hospital, Phoenix, Arizona, United States; 7 International Center for Cardiovascular Interventions, Heart Center Bonn, Medizinischen Klinik und Poliklinik II, Bonn, Germany; 8 Städtisches Klinikum Dresden, Medizinische Klinik, Dresden, Germany

**Keywords:** venous thrombosis, deep vein thrombosis, pulmonary embolism

## Abstract

**Objectives** In patients with suspected venous thromboembolism (VTE), the D-dimer assay is commonly utilized as part of the workup. The assay is primarily used to determine whether to proceed with radiographic imaging. We compared D-dimer levels in patients suspected of having VTE. We hypothesized that higher D-dimer values predict a higher likelihood of subsequent VTE diagnosis.

**Methods** We conducted a secondary analysis of a multinational, prospective observational study of low- to intermediate-risk adult patients presenting to the emergency department with suspicion of VTE. Demographic and clinical data were collected in a structured manner. Advanced imaging including ultrasound, computed tomography (CT) pulmonary angiography, and ventilation/perfusion scanning was obtained at the discretion of the treating physicians. Imaging was evaluated by board-certified radiologists in real time. D-dimer values' bins were evaluated using a logistic regression model.

**Results** We evaluated 1,752 patients for suspected deep vein thrombosis (DVT), with 191 (10.4%) DVT positive. We evaluated 1,834 patients for suspected pulmonary embolism (PE), with 108 (5.9%) PE positive. Higher D-dimer values in both groups were associated with higher likelihood of subsequent VTE diagnosis, with D-dimer values > 3,999 ng/mL in both groups having the highest incidence of VTE. More than 50% of those patients were VTE positive.

**Conclusions** Increasing D-dimer values predict increased likelihood of being found VTE positive in this patient population. Among those in the highest D-dimer category, > 3,999 ng/mL, over half of patients were VTE positive. Further research could determine additional nuance in D-dimer as a tool to work up suspected VTE.

## Introduction

D-dimer testing in the workup of patients with potential venous thromboembolism (VTE) is commonplace. 1 2 3 4 5 6 7 When used appropriately, D-dimer testing has demonstrated a reduction in radiographic imaging, decreased emergency department (ED) length of stay, and decreased total health care costs in appropriately risk-stratified groups. 8 However, some studies have demonstrated an increase in workups and radiographic imaging for VTE with the introduction of the D-dimer assay without an associated increase in diagnosis. 9

Since its implementation, the D-dimer has been utilized as a dichotomous test. When used in this manner, the D-dimer has a specificity at or below 50%. 1 10 Studies in recent years have sought to adjust dichotomous D-dimer thresholds based on different criteria such as: age, clinician's pretest probability, or pregnancy. 11 12 13 In each case, however, the D-dimer result is still used dichotomously; that is, below a certain threshold the test is considered negative and above the threshold it is considered positive. There have been several studies demonstrating that D-dimer values can be used as continuous variables to predict likelihood of pulmonary embolism (PE). These studies have shown that a patient's chance of having PE increases with rising D-dimer concentration. 14 15 16 However, there have not been any studies to date evaluating use of continuous D-dimer values in patients with suspected deep vein thrombosis (DVT). One study found a D-dimer value greater than 3.6 μg/mL increased the likelihood of subsequent DVT diagnosis. 17 To date, there have not been any studies specifically examining the upper value D-dimer concentrations in the risk stratification of patients with suspected DVT.

In this secondary analysis of a prospective observational study of a D-dimer assay performed at multiple centers across the United States and Europe, there are two hypotheses. First, we hypothesize that the likelihood of DVT and PE increases linearly with rising D-dimer values. Second, using a bayesian approach, there are D-dimer values above which a single negative radiographic interrogation (ultrasound, computed tomography pulmonary angiography [CTPA], or ventilation/perfusion [VQ]) no longer has sufficient posttest probably to avoid further evaluation in select cases using current recommended guidelines. 18

## Methods

### Setting

We performed a secondary analysis of a prospective, observational study of D-dimer testing in consecutive ED patients with suspected VTE from 23 centers (17 USA, 6 Europe). 19 The primary study was performed to determine the test characteristics of two available D-dimer assays (VIDAS D-dimer and Innovance D-dimer) using standard and age-adjusted cutoffs. All participating centers had the capability for CTPA, VQ scanning, venous ultrasound, and peripheral venography. Siemens Healthcare Diagnostics (Newark, Delaware, United States) was the sponsor of the study. Siemens had no role in analyzing or interpreting the data. The institutional review board (IRB) of each participating institution approved the study prior to participation and enrollment of any subjects.

### Selection of Participants

Eligible individuals were 18 years and older who presented to an ED or outpatient clinic with suspected VTE and received objective testing by the treating clinician. D-dimer samples using the Innovance D-dimer assay were drawn after the decision was made by the clinician to test for DVT or PE but prior to results of the diagnostic workup. These individuals needed to be capable of providing informed consent. As part of their usual medical care, all participants received objective testing for VTE. Patients with possibility of recurrent VTE were included in the study. We excluded patients if they had high pretest probability for VTE (Wells' PE score > 6 or Wells' DVT score ≥ 2). Our other exclusion criteria included pregnancy and anticoagulation use for > 24 hours prior to blood sample collection. We obtained written consent for all patients by trained research staff.

### Data Collection

After patient consent, we collected baseline demographic data, including gender, race, age, Wells' pretest probability score for DVT or PE, D-dimer value, result of imaging procedure(s), outcome at 3-month follow-up, and final diagnosis. The data were collected by trained research staff. Resident and attending physicians gathered the data and calculated the Wells DVT and Wells PE scores to generate a pretest probability. We collected blood samples and pretest probability was assessed before the patient's usual care diagnostic test results had returned. Blood samples were drawn into 3.2% sodium citrate tubes. Within 4 hours of collection, samples were centrifuged to platelet-free plasma, transferred to microtubes, and frozen to ≤ −70°C. The D-dimer concentrations used for this study were measured by the Innovance D-dimer platform (Siemens Healthcare Diagnostics, Newark, Delaware, United States) on the CS-5100 system at a central laboratory. D-dimer tests ordered as part of usual clinical care were not used for this analysis to maintain consistency across centers.

### Outcome Measures

Participants underwent a structured evaluation for VTE consistent with local usual care protocols. We considered patients to have a PE if their CTPA demonstrated a filling defect in a pulmonary artery or if a VQ scan was read as high probability for PE. If a patient was low or intermediate pretest probability for VTE but did not have imaging performed but had a negative D-dimer performed by the hospital's clinical laboratory, we considered them to have ruled out for VTE. We considered patients to have a DVT if a thrombus in a deep venous leg vein proximal to, or at the level of, the calf was found by either venous ultrasound or contrast venography. Patients with possibility of recurrent VTE were included in the study. For those patients, they were considered to be PE or DVT positive if imaging demonstrated clot in a new location or extension of a previously documented DVT. All radiologic studies were interpreted by board-certified radiologists.

The D-dimer categories were decided after analyzing the total number of patients included in specific “bins.” By maintaining “bins” of 1,000 ng/mL beyond a D-dimer concentration of 1,000 ng/mL, we were able to maintain an appropriate number of patients to reduce the risk of random chance causing the outcome. We decided > 3,999 ng/mL was the upper level “bin” so as to maintain an appropriate number of patients in each group.

Three months after the index visit, we performed a phone call follow-up with patients and we also reviewed their medical records. Patients with an initial negative workup for VTE were considered to have a PE or DVT during their 3-month follow-up if they reported a diagnosed DVT or PE in the interval. Patients were considered lost to follow-up if they did not respond to five follow-up phone calls. For analysis purposes, these patients were not considered to have hemodynamically significant DVT or PE as long as they had negative criterion standard in their index visit.

### Statistical Analysis

Demographic data are reported as means with standard deviations (SD) or simple percentages. Standard methods were utilized for calculating characteristics of D-dimer values such as odds ratios. Patients were analyzed in D-dimer bins of the following values: a logistic regression model was constructed using D-dimer level as predictor variable and VTE occurrence as outcome, while controlling for several variables. In patients with DVT, we controlled for gender, race, and the individual components of the Wells DVT score. In patients with PE, we controlled for age as well as the individual components of Wells' PE score. Data were analyzed using SAS version 9.4 (SAS Institute, Cary, North Carolina, United States). Adjusted odds ratios (ORs) were utilized for the D-dimer bins because they were derived from multivariable components as noted above.

## Results

**Fig. 1:**
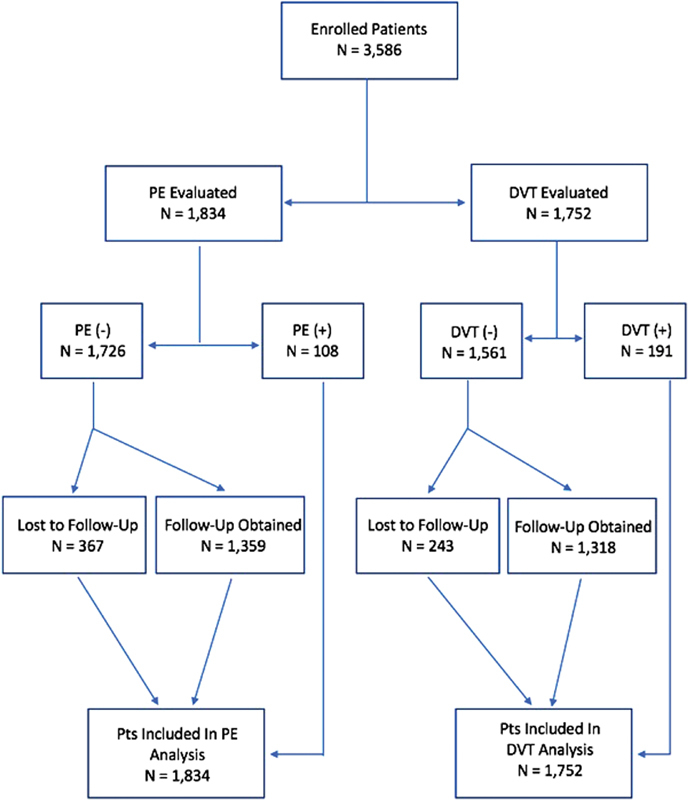
Enrollment.

In total, 3,586 patients were evaluated for VTE; of these, 1,752 were evaluated for DVT and 1,834 were evaluated for PE ( Fig. 1 ). Among patients evaluated for DVT, the DVT prevalence was 10.9% (191/1752). Seventy-eight (4.4%) had a calf DVT, and 113 (6.4%) had a proximal DVT. Of the 191 DVT-positive patients, 4 (0.2%) patients were considered to be DVT negative on their index visit and were subsequently found to be DVT positive at follow-up. The mean age was 53.1 ± 16.2 years, and 710 (40.5%) were male.

There were 743 patients in the DVT cohort with a D-dimer < 500 ng/mL. Eighteen (2.4%) of these were found to be DVT positive. The average Wells' DVT score in these patients was 0.78 (SD 1.06). Of the 18 patients that were DVT positive, 9 had an isolated calf DVT. All 18 were diagnosed on the index visit. Nine out of 18 (50%), had a prior history of DVT by Wells' DVT score.

Among patients evaluated for PE, the PE incidence was 5.9% (108/1834). In total, 101 (5.5%) had a segmental or larger PE on imaging. Seven (0.4%) were found to have a subsegmental PE. Of the 108 PE-positive patients, 7 (0.4%) patients were considered to be PE negative on their index visit and were subsequently found to be PE positive at follow-up. The mean age was 47.4 ± 15.8 years, and 676 (36.9%) were male. Additional demographic information is given in Table 1 . For the purposes of this analysis, those lost to follow-up were presumed to be VTE negative.

**Table 1:** Characteristics of patients evaluated for VTE

	DVT ( *N* = 1,752)			PE ( *N* = 1,834)		
	*N*	%	SD	*N*	%	SD
Age (mean)	–	53.1	16.2	–	47.7	15.8
Gender (male)	710	40.5	–	676	36.9	–
Race						
White	1,172	66.9	–	1,081	58.9	–
Black	475	27.1	–	553	30.2	–
Hispanic	79	4.5	–	145	7.9	–
Other	14	0.8	–	37	2.0	–
Asian	12	0.7	–	18	1.0	–
Wells' score category						
Low	576	32.9	–	1,175	64.1	–
Intermediate	559	31.9	–	658	35.9	–
Unknown	617	35.2	–	1	0.1	–

There were 962 patients in the PE cohort with a D-dimer < 500 ng/mL. Three (0.3%) of these were found to be PE positive. In those PE patients, the average Wells' PE score was 2.33. This is considered an intermediate-risk Wells' PE score. All three were diagnosed on their index visit.

**Fig. 2:**
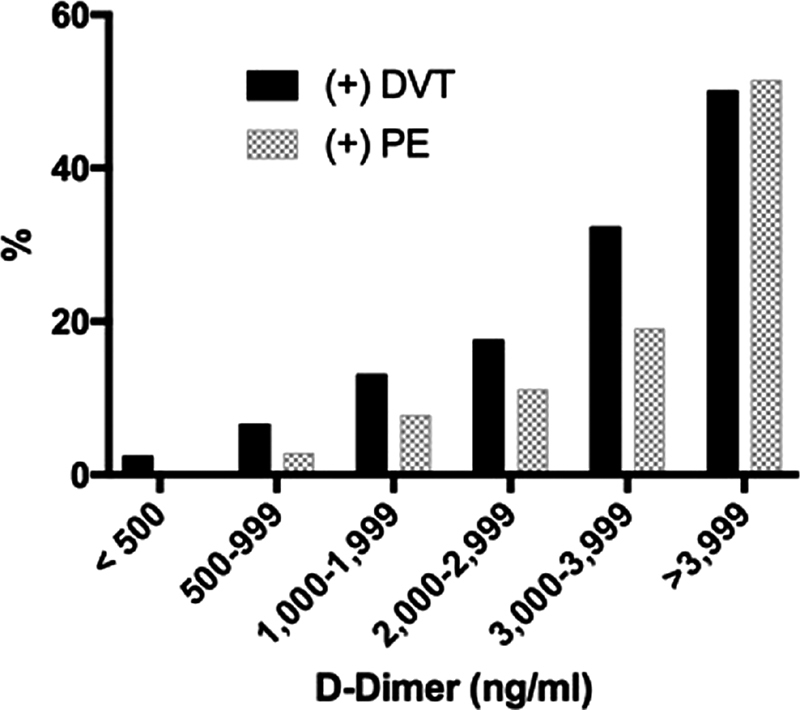
DVT- and PE-positive patients based on D-dimer values.

D-dimer values were obtained on all patients. Table 2 shows that the OR for DVT rises with progressively higher D-dimer concentrations to a maximum of 52 (95% confidence interval [CI], 27–99) in patients with a D-dimer > 3,999 ng/mL. Utilizing a logistic regression model, the adjusted OR for D-dimer value in DVT as a continuous variable was 1.34 (95% CI, 1.26–1.42), *p* < 0.0001. The proportion of DVT diagnoses by D-dimer category is shown in Fig. 2 .

**Table 2:** Proportion of DVT-positive and DVT-negative patients by D-dimer result

D-dimer (ng/mL)	Patients (#)	Percentage	aOR (95% CI)
<500			
DVT (+)	18	2.4	Reference
DVT (−)	725	97.6	
500–999			
DVT (+)	28	6.5	3.3 (1.7–6.1)
DVT (−)	401	93.5	
1,000–1,999			
DVT (+)	34	13.0	6.9 (3.7–12.8)
DVT (−)	228	87.0	
2,000–2,999			
DVT (+)	20	17.5	10.8 (5.2–22.4)
DVT (−)	94	82.5	
3,000–3,999			
DVT (+)	20	32.3	24.6 (11.2–54.3)
DVT (−)	42	67.7	
>3,999			
DVT (+)	71	50.0	51.7 (26.9–99.2)
DVT (−)	71	50.0	

Table 3 shows that the OR for PE rises with progressively higher D-dimer concentrations, to a maximum of 221 (95% CI, 65–752) in patients with a D-dimer >3,999 ng/mL. At the highest D-dimer values, more than half of the patients were ultimately diagnosed with VTE. Using a logistic regression model, the adjusted OR for D-dimer value in PE as a continuous variable was 1.31 (95% CI, 1.23–1.40), *p* < 0.0001. The rates of PE versus D-dimer in the various numerical categories are shown in Fig. 2 .

**Table 3:** Proportion of PE-positive and PE-negative patients by D-dimer result

D-dimer (ng/mL)	Patients (#)	Percentage	aOR (95% CI)
<500			
PE (+)	3	0.3	Reference
PE (−)	959	99.7	
500–999			
PE (+)	11	2.8	7.2 (2.0–26.1)
PE (−)	387	97.2	
1,000–1,999			
PE (+)	18	7.7	17.7 (5.1–61.7)
PE (−)	217	92.3	
2,000–2,999			
PE (+)	13	14.4	37.4 (10.2–138.1)
PE (−)	77	85.6	
3,000–3,999			
PE (+)	8	19.0	42.4 10.2–175.9)
PE (−)	34	81.0	
>3,999			
PE (+)	55	51.4	221.5 (65.2–753.0)
PE (−)	52	48.6	

**Fig. 3:**
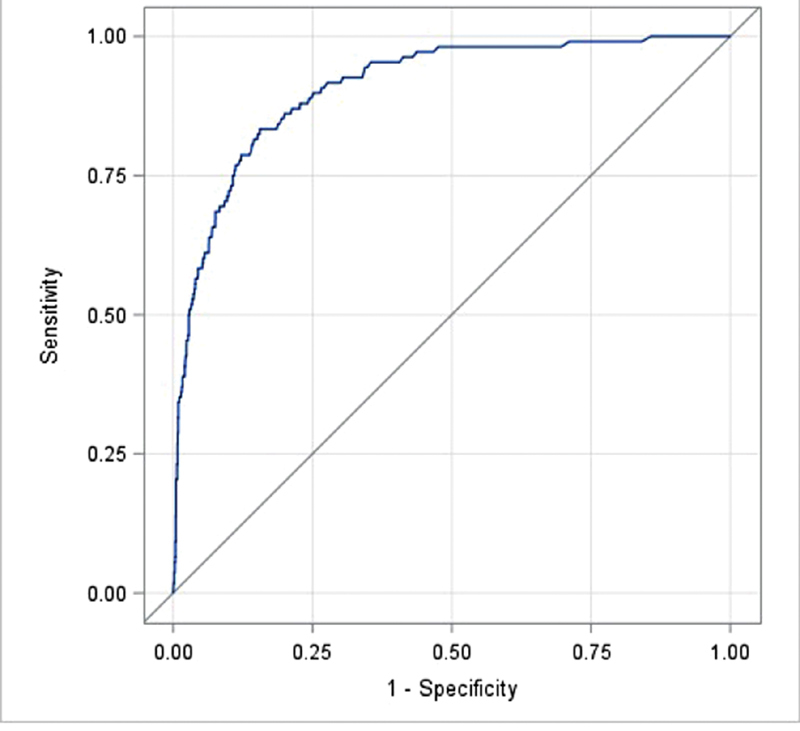
ROC curve for D-dimer with regard to likelihood of PE. Area under curve is 0.910.

**Fig. 4:**
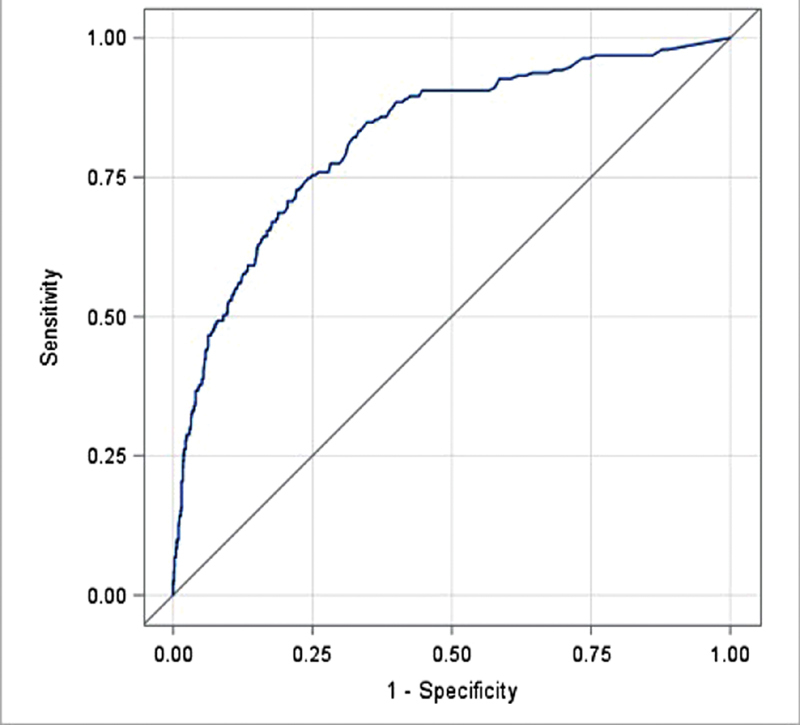
ROC curve for D-dimer in patients with suspected DVT. The area under the curve was 0.823.

Figs. 3 and 4 demonstrate the receiver operating characteristic (ROC) curves generated for patients with suspected PE and DVT, respectively, when utilizing the D-dimer as an initial test. The area under the curve for both PE and DVT was high, 0.910 (0.882–0.937) and 0.823, respectively.

In the highest D-dimer category, > 3,999 ng/mL, by eliminating patients with active cancer and/or recent surgery/bedridden by Wells' DVT criteria, we found 52/95 patients, 54.7% (95% CI, 44.2–64.9%), to be DVT positive. In the patients with suspected PE in the highest D-dimer category, eliminating those with treated malignancy and/or with immobility or recent surgery by Wells' PE criteria left 38/67 patients, 56.7% (95% CI, 44.1–68.6%), PE positive.

## Discussion

In this multicenter international observational study, we observed an increasing rate of VTE with increasing D-dimer levels. There appears to be value in utilizing the D-dimer in a more nuanced approach to VTE workup beyond its current dichotomous use. There does not appear to be an “absolute cutoff” beyond which meaningful information is no longer provided. Future decision instruments may be able to incorporate the D-dimer value as a spectrum rather than a dichotomous cutoff in developing pretest probabilities for patients with suspected VTE.

Patients with a D-dimer value < 500 ng/mL had clinically apparent DVT and PE prevalence rates of 2.4 and 0.3%, respectively. Given the risks of radiation, increased length of stays, and increased costs associated with additional workups, 8 the data support no further VTE workup if the D-dimer returns with a normal value. It is important to recognize that patients with appropriately low pretest probability for PE should undergo D-dimer testing to reduce unnecessary radiation exposure. Studies have shown that D-dimer testing is bypassed in patients that meet criteria for initial D-dimer testing as part of the workup in favor of radiographic imaging. 20 The data demonstrated here reiterate that individuals who are below the threshold D-dimer value of < 500 ng/mL should have their workup for VTE ceased if the test was ordered in the appropriate risk stratification group.

In this study, we found a clear relationship between increasing D-dimer values and likelihood of VTE diagnosis. This is the first study to evaluate this connection in patients with suspected DVT. In those patients at the highest D-dimer values (>3,999 ng/mL in our study), over 50% were found to be VTE positive. With the known false-negative rate for both DVT ultrasound and CTPA or VQ imaging for PE, clinicians should consider additional workup in those patients with D-dimer values in this range and either nondiagnostic or negative initial testing. 21 22 Empiric anticoagulation has been recommended by some experts for patients initially deigned high risk for VTE prior to completion of imaging studies. 23

In the > 3,999 ng/mL cohort of PE-positive patients, if we take the reported sensitivities and specificities of CTPA and VQ as noted in PIOPED II, we are able to calculate a negative likelihood ratio for both studies. 24 CTPA has a sensitivity of 83% and a specificity of 96%. VQ has a sensitivity of 77.4% and a specificity of 97.7%. The negative likelihood ratios of CTPA and VQ are therefore 0.177 and 0.188, respectively. With the prevalence of disease in the highest D-dimer cohort being 50%, therefore, a negative imaging study of either CTPA or VQ scanning leads to posttest probabilities of 15% for CTPA and 18.8% for VQ scan. For the clinician at the bedside, this means that one has to be attentive to the individual patient and recognize the risk of false-negative imaging findings in this group. The high sensitivity and specificity of venous ultrasound for proximal DVT, 96.5 and 94.3%, respectively, 25 reveal that negative imaging studies have a much lower posttest probability in the highest D-dimer cohort in those suspected of DVT. A presumed prevalence of 50% in the > 3,999 ng/mL category of D-dimer coupled with a negative likelihood ratio of 0.036 for venous ultrasound leads to a posttest probability of 3.6% with a negative DVT ultrasound.

Our dataset presents the spectrum of patients with suspected VTE at low and intermediate pretest probability. While much scrutiny has focused of the lower D-dimer levels, the cohort of patients with high D-dimer presents different challenges in risk stratification. When patients initially thought to be at low or moderate pretest probability for PE subsequently have high D-dimer values > 3,999 ng/mL, the diagnostic strategy still includes CTPA, VQ, or venous duplex ultrasonography. However, if these studies are negative or nondiagnostic, consideration should be given to further testing. One possibility is that the ultrasound or CTPA is falsely negative. As such, additional testing with angiography or perfusion scintigraphy may be options. Alternatively, the location of the thrombosis may be in an atypical location such as the pelvic, central nervous system, upper extremities, or jugular vessels. Additional consideration should be made to starting empiric anticoagulation therapy given the probability of disease in this patient cohort.

In the highest D-dimer category, eliminating patients with suspected DVT who were positive on their Wells' DVT score for recent bedbound/surgery and/or active cancer revealed a prevalence of disease > 50% in this cohort. Eliminating patients with suspected PE in the highest D-dimer category with a Wells PE score positive for active cancer and/or recent surgery/immobilization also revealed a prevalence of disease > 50%. These data support that in these patients with two common causes of D-dimer elevation to be eliminated, the prevalence of disease remains high. VTE is the most common etiology of D-dimer elevation to that level in this group of patients.

While D-dimer values <500 ng/mL have been shown to be highly sensitive for ruling out VTE, the test is not perfect and false-negatives can occur. In the primary study performed, a negative D-dimer had a sensitivity of 98% for PE and 92% for DVT. 19 False-negatives have been associated with increased age of thrombus. One study found subsequent D-dimer values reaching 25% of initial value of D-dimer VTE by 1 week of symptoms. 26 Another recommended not utilizing D-dimer testing if clinical symptoms have lasted for greater than 1 week. 27 A prior study suggested that fibroblasts invade old thrombi over time. As a result, fibrin is collagenized and becomes poorly degradable using fibrinolytic enzymes. 28 Our analysis did not include duration of symptoms as part of data collection. Prior treatment with anticoagulation has also been associated with false-negative D-dimer values. There is also the possibility that normal D-dimer levels may be the result of an existing dysfibrinogenemia such as Dusard's syndrome, where the clot formed is poorly degradable, and thus has low D-dimer values. 29 30

In the 18 patients who had a D-dimer value < 500 ng/mL, but were DVT positive, 9 were found to have an isolated calf DVT. Prior literature has demonstrated that in patients with isolated calf DVTs, the sensitivity and specificity of the Wells DVT score was low at 47 and 74%, respectively. Compared with DVT-negative patients, however, patients with isolated calf DVTs had higher D-dimer levels. 31 The diagnosis of isolated calf DVT remains controversial, however.

## Limitations

There were several limitations to this study. First, there were a significant number of patients lost to follow-up after their initial evaluation for VTE. For those found to be PE negative on their index visit, 367 (21.1%) were lost to follow-up. In those found DVT negative on their index visit, 243 (14.8%) were lost to follow-up. While every effort was made to limit this number with five phone calls made to attempt follow-up, nonetheless their ultimate outcomes are not known.

We made the decision to denote isolated subsegmental PE and calf DVT as being VTE positive for the purposes of data analysis. This is an area of controversy as some data support these entities as being false-positive or not needing anticoagulation. 32 33 Other studies consider these to be VTE events. 24 25

We used the Innovance D-dimer as our benchmark. While each D-dimer assay has a slightly different performance characteristics, this was likely not a large factor in outcomes, given the high concordance of the Innovance system with the VIDAS D-dimer system. 34

Finally, due to the small sample size of some of the D-dimer categories, there are wide CIs in some of the groups. We cannot be sure of the specific ORs within each category, but can draw general conclusions given the increasing adjusted ORs.

## Future Research and Implications

This study brings to the forefront the application of the D-dimer in the workup of patients with suspected VTE. Currently used as a primarily dichotomous yes/no test as to whether or not to proceed in VTE evaluation, the association between D-dimer value and likelihood of VTE diagnosis suggests that there is room for subtlety in its application. Our results allow the use of simple bayesian calculators, which allow clinicians to more accurately and specifically estimate a patient's pretest and posttest probabilities of VTE based on D-dimer and imaging results.

Future research can evaluate the patients who are false-negatives when D-dimer is utilized in suspected VTE. Evaluation of the possible age of thrombus on D-dimer values can be one avenue to explore. It is also possible these patients have abnormal fibrinolytic patterns such as increased levels of α _2_ -antiplasmin or have fibrin that is poorly degradable such as in Dusard's syndrome. Additional studies can be aimed toward determining susceptibility of clot formed by clotting plasma in vitro to degradability by fibrinolytic enzymes.

Future research seeking to incorporate D-dimer values or ranges of values into decision instruments may refine the clinician's approach to suspected VTE that increases sensitivity of detecting disease while maintaining appropriate specificity.

## References

[1] Stein P D Hull R D Patel K C D-dimer for the exclusion of acute venous thrombosis and pulmonary embolism: a systematic review Ann Intern Med 2004 140 08 589 602 15096330 10.7326/0003-4819-140-8-200404200-00005

[2] Kline J A Nelson R D Jackson R E Courtney D M Criteria for the safe use of D-dimer testing in emergency department patients with suspected pulmonary embolism: a multicenter US study Ann Emerg Med 2002 39 02 144 152 11823768 10.1067/mem.2002.121398

[3] Oudega R Moons K G Hoes A W Ruling out deep venous thrombosis in primary care. A simple diagnostic algorithm including D-dimer testing Thromb Haemost 2005 94 01 200 205 16113804 10.1160/TH04-12-0829

[4] Ilkhanipour K Wolfson A B Walker H Combining clinical risk with D-dimer testing to rule out deep vein thrombosis J Emerg Med 2004 27 03 233 239 15388207 10.1016/j.jemermed.2004.04.010

[5] Kristoffersen A H Ajzner E Rogic D Is D-dimer used according to clinical algorithms in the diagnostic work-up of patients with suspicion of venous thromboembolism? A study in six European countries Thromb Res 2016 142 1 7 27085136 10.1016/j.thromres.2016.04.001

[6] Kabrhel C Van Hylckama Vlieg A Muzikanski A Multicenter evaluation of the YEARS criteria in emergency department patients evaluated for pulmonary embolism Acad Emerg Med 2018 25 09 987 994 29603819 10.1111/acem.13417

[7] McLenachan C J Chua O Chan B S Vecellio E Chiew A L Comparison of Wells and YEARS clinical decision rules with D-dimer for low risk pulmonary embolus patientsIntern Med J 2018; doi: 10.1111/imj.14138 10.1111/imj.14138 30324677

[8] Duriseti R S Brandeau M L Cost-effectiveness of strategies for diagnosing pulmonary embolism among emergency department patients presenting with undifferentiated symptoms Ann Emerg Med 2010 56 04 321 3.32E12 20605261 10.1016/j.annemergmed.2010.03.029 PMC3699695

[9] Kabrhel C Matts C McNamara M Katz J Ptak T A highly sensitive ELISA D-dimer increases testing but not diagnosis of pulmonary embolism Acad Emerg Med 2006 13 05 519 524 16551779 10.1197/j.aem.2005.12.012

[10] Carrier M Righini M Djurabi R K VIDAS D-dimer in combination with clinical pre-test probability to rule out pulmonary embolism. A systematic review of management outcome studies Thromb Haemost 2009 101 05 886 892 19404542

[11] Murphy N Broadhurst D I Khashan A S Gilligan O Kenny L C O'Donoghue K Gestation-specific D-dimer reference ranges: a cross-sectional study BJOG 2015 122 03 395 400 24828148 10.1111/1471-0528.12855

[12] Righini M Van Es J Den Exter P L Age-adjusted D-dimer cutoff levels to rule out pulmonary embolism: the ADJUST-PE study JAMA 2014 311 11 1117 1124 24643601 10.1001/jama.2014.2135

[13] van der Hulle T Cheung W Y Kooij S Simplified diagnostic management of suspected pulmonary embolism (the YEARS study): a prospective, multicentre, cohort study Lancet 2017 390(10091):289 297 28549662 10.1016/S0140-6736(17)30885-1

[14] Tick L W Nijkeuter M Kramer M H High D-dimer levels increase the likelihood of pulmonary embolism J Intern Med 2008 264 02 195 200 18452520 10.1111/j.1365-2796.2008.01972.x

[15] Kohn M A Klok F A van Es N D-dimer interval likelihood ratios for pulmonary embolism Acad Emerg Med 2017 24 07 832 837 28370759 10.1111/acem.13191

[16] Shah K Quaas J Rolston D Magnitude of D-dimer matters for diagnosing pulmonary embolus Am J Emerg Med 2013 31 06 942 945 23685058 10.1016/j.ajem.2013.03.009

[17] Nata S Hiromatsu S Shintani Y Ohno T Akashi H Tanaka H D-dimer value more than 3.6 μg/ml is highly possible existence deep vein thrombosis Kurume Med J 2013 60 02 47 51 24464132 10.2739/kurumemedj.ms61009

[18] Wolf S J Hahn S A Nentwich L M Raja A S Silvers S M Brown M D; American College of Emergency Physicians Clinical Policies Subcommittee (Writing Committee) on Thromboembolic Disease.Clinical Policy: Critical issues in the evaluation and management of adult patients presenting to the emergency department with suspected acute venous thromboembolic disease Ann Emerg Med 2018 71 05 e59 e109 29681319 10.1016/j.annemergmed.2018.03.006

[19] Parry B A Chang A M Schellong S M International, multicenter evaluation of a new D-dimer assay for the exclusion of venous thromboembolism using standard and age-adjusted cut-offs Thromb Res 2018 166 63 70 29656169 10.1016/j.thromres.2018.04.003

[20] Yan Z Ip I K Raja A S Gupta A Kosowsky J M Khorasani R Yield of CT pulmonary angiography in the emergency department when providers override evidence-based clinical decision support Radiology 2017 282 03 717 725 27689922 10.1148/radiol.2016151985 PMC5330300

[21] Hutchinson B D Navin P Marom E M Truong M T Bruzzi J F Overdiagnosis of pulmonary embolism by pulmonary CT angiography AJR Am J Roentgenol 2015 205 02 271 277 26204274 10.2214/AJR.14.13938

[22] Markel A Weich Y Gaitini D Doppler ultrasound in the diagnosis of venous thrombosis Angiology 1995 46 01 65 73 7818159 10.1177/000331979504600109

[23] Hogg K E Brown M D Kline J A Estimating the pretest probability threshold to justify empiric administration of heparin prior to pulmonary vascular imaging for pulmonary embolism Thromb Res 2006 118 05 547 553 16356538 10.1016/j.thromres.2005.11.003

[24] Stein P D Fowler S E Goodman L R Multidetector computed tomography for acute pulmonary embolism N Engl J Med 2006 354 22 2317 2327 16738268 10.1056/NEJMoa052367

[25] Goodacre S Sampson F Thomas S van Beek E Sutton A Systematic review and meta-analysis of the diagnostic accuracy of ultrasonography for deep vein thrombosis BMC Med Imaging 2005 5 6 16202135 10.1186/1471-2342-5-6 PMC1262723

[26] D'Angelo A D'Alessandro G Tomassini L Pittet J L Dupuy G Crippa L Evaluation of a new rapid quantitative D-dimer assay in patients with clinically suspected deep vein thrombosis Thromb Haemost 1996 75 03 412 416 8701399

[27] Goldin Y Pasvolsky O Rogowski O The diagnostic yield of D-dimer in relation to time from symptom onset in patients evaluated for venous thromboembolism in the emergency medicine department J Thromb Thrombolysis 2011 31 01 1 5 20419335 10.1007/s11239-010-0480-6

[28] Mirshahi M Azzarone B Soria J Mirshahi F Soria C The role of fibroblasts in organization and degradation of a fibrin clot J Lab Clin Med 1991 117 04 274 281 2010668

[29] Soria J Soria C Caen P A new type of congenital dysfibrinogenaemia with defective fibrin lysis--Dusard syndrome: possible relation to thrombosis Br J Haematol 1983 53 04 575 586 6830701 10.1111/j.1365-2141.1983.tb07309.x

[30] Lijnen H R Soria J Soria C Collen D Caen J P Dysfibrinogenemia (fibrinogen Dusard) associated with impaired fibrin-enhanced plasminogen activation Thromb Haemost 1984 51 01 108 109 6539000

[31] Sartori M Cosmi B Legnani C The Wells rule and D-dimer for the diagnosis of isolated distal deep vein thrombosis J Thromb Haemost 2012 10 11 2264 2269 22906051 10.1111/j.1538-7836.2012.04895.x

[32] Kearon C Akl E A Ornelas J Antithrombotic therapy for VTE disease: CHEST Guideline and Expert Panel Report Chest 2016 149 02 315 352 26867832 10.1016/j.chest.2015.11.026

[33] Palareti G Do isolated calf deep vein thrombosis need anticoagulant treatment? J Thorac Dis 2016 8 12 E1691 E1693 28149615 10.21037/jtd.2016.12.93 PMC5227197

[34] Salvagno G L Lippi G Manzato F Analytical comparison of AxSYM, HemosIL DD HS and Innovance D-dimer immunoassays with the Vidas D-dimer Int J Lab Hematol 2009 31 04 475 477 18503567 10.1111/j.1751-553X.2008.01077.x

